# Timing of poverty in childhood and adolescent health: Evidence from the US and UK

**DOI:** 10.1016/j.socscimed.2017.12.004

**Published:** 2018-01

**Authors:** Michael J. Green, Haley Stritzel, Chelsea Smith, Frank Popham, Robert Crosnoe

**Affiliations:** a MRC/CSO Social and Public Health Sciences Unit, University of Glasgow, United Kingdom; b Department of Sociology and Population Research Center, University of Texas at Austin, United States

**Keywords:** Adolescent health, Smoking, Life course, Income, Poverty, Cross-national

## Abstract

Childhood poverty is associated with poorer adolescent health and health behaviours, but the importance of the timing of poverty remains unclear. There may be critical or sensitive periods in early life or early adolescence, or poverty may have cumulative effects throughout childhood. Understanding when poverty is most important can support efficient timing of interventions to raise family income or buffer against the effects of low income, but answers may vary across social contexts. The US and the UK are a useful comparison with similar liberal approaches to cash transfers, but very different approaches to healthcare provision. Utilising data from large population studies in the US (n = 9408; born 1979–1996) and UK (n = 1204; born 1991–1997), this study employs a structured life course approach to compare competing hypotheses about the importance of the timing or pattern of childhood exposure to poverty in predicting adolescent health limitations, symptoms of psychiatric distress, and smoking at age 16 (age 15/16 in US). Household income histories identified experience of poverty (measured as <60% of the national median equivalised income for a given year) in early life (ages 0–5), mid-childhood (ages 6–10) and early adolescence (ages 11–15). The Bayesian Information Criterion (BIC) compared fit across models with variables representing different life course patterns of exposure to poverty. Adolescent distress was not associated with poverty in either country. In both countries, however, variables representing cumulative or persistent experiences of poverty exhibited optimal fit of all poverty exposure variables in predicting adolescent smoking and health limitations. There was also evidence of an early life sensitive period for smoking in the US. Poverty was more persistent in the US, but associations between poverty and outcomes were consistent across countries. Although poverty can have cumulative effects on health and behaviour, early interventions may offer the best long-term protection.

## Highlights

• Poverty in childhood was associated with adolescent smoking and health limitations.
• Poverty in childhood was not associated with adolescent mental health.
• Indicators of cumulative or persistent poverty best predicted adolescent outcomes.
• Poverty was more persistent in the US than the UK.
• Poverty showed similar associations with adolescent health in the US and UK.

## 1 Background

There is growing recognition of the importance of adolescence for shaping health over the life course (Due et al., 2011, Sawyer et al., 2012, Viner et al., 2015). First, adolescence is a key stage in the development of health behaviours, such as smoking, and habits developed during this stage of life often persist into adulthood (Due et al., 2011, Sawyer et al., 2012, Viner et al., 2012). Second, adolescence is a time of rapid physical, social, emotional and cognitive development, so health conditions and behaviours that affect or reflect developmental processes during adolescence can have long term consequences (Due et al., 2011, Sawyer et al., 2012, Viner et al., 2012, Viner et al., 2015). Finally, poor physical or mental health in adolescence may present challenges to educational and occupational success, which can have lasting impacts on young people's life chances (Haas, 2006, Miech et al., 1999, Sweeting et al., 2016). One potentially key determinant of adolescent health and behaviour is the socioeconomic status (SES) of the household in which the young person grew up. Children in more disadvantaged households tend to have poorer outcomes (Chen et al., 2006, Due et al., 2011, Emerson et al., 2005, Hanson and Chen, 2007, Joinson et al., 2016, McLeod and Shanahan, 1993), although some studies suggest that adolescence can be a period of relative equality in health (Siahpush and Singh, 2000, Sweeting et al., 2016, West et al., 1990), if not behaviour (Green et al., 2016). This study focuses on associations between childhood SES and two adolescent health outcomes (physical health limitations and mental distress), and a health behaviour (smoking).

SES is a broad and heterogeneous concept measured with a range of indicators, such as occupation, education, and income that can have different meanings and different implications for intervention (Galobardes et al., 2006, Krieger et al., 1997, Liberatos and Link, 1988). An oft-cited study by Duncan et al. (1998) linked household income to children's educational outcomes (as well as teen pregnancy) in the US, showing a non-linear association with particularly strong disadvantages for those in the lowest income households. Importantly, they asserted it may be easier to design programmes that alter family income (e.g. via welfare benefits, tax credits, etc.) than to alter other family characteristics such as parental education or occupation. Thus, with a view to informing potential intervention strategies, the focus of this study is the nature of the association between childhood income poverty and health-related outcomes in adolescence. There are many ways to define poverty (Hagenaars and de Vos, 1988, Wagle, 2002), and we opt for a commonly-used, relative definition (<60% of the national median household equivalised income). This means that the focus is on having a low income relative to other households in a country within a given year, rather than on the absolute level of resources available. Compared to an absolute measure, such a definition has the advantage of a similar meaning in terms of relative deprivation when comparing across countries and over time. In investigating the effects of such poverty, we follow examples from life course research—including several studies linking childhood poverty to educational outcomes—in acknowledging that effects may be dependent on the timing, duration and sequencing of exposure to poverty (Mishra et al., 2009, Wagmiller et al., 2006). Doing so can help identify the most effective points to intervene, which is important considering the costs associated with any intervention to raise family income or buffer its deleterious effects.

Life course epidemiology, for example, often contrasts models of accumulation, where exposure effects depend on the duration of exposure and are independent of timing, with critical or sensitive period models, where exposure effects depend on the timing of exposure (Ben-Shlomo and Kuh, 2002, Ben-Shlomo et al., 2014, Green and Popham, 2017). The term ‘critical period’ denotes a period of exclusive risk, while ‘sensitive period’ denotes a period of heightened risk. Duncan et al. investigated timing by comparing effects of income in early life (ages 0–5), mid-childhood (ages 6–10) and early adolescence (ages 11–15) and showed that poverty experienced during early life was a sensitive period for the relationship between poverty and poorer academic achievement (Duncan et al., 1998). Early life may also be a sensitive period in determining adolescent health. Many have argued, much as some now do for adolescence, that adversities during the rapid physical, social, emotional and cognitive development of early life can have lasting impacts (Marmot, 2010, Sawyer et al., 2012, Shonkoff and Garner, 2012), and these impacts may lead to greater propensities for poor health and behaviours in adolescence. Evidence for particularly sensitive periods in early life may therefore indicate that income acts on adolescent health via impacts on development (in addition to other mechanisms not specific to early life). Evidence for early life as a critical period could indicate that income operates to shape health exclusively via such developmental mechanisms.

The early life critical or sensitive period hypotheses described above might be contrasted against other models for the impact of exposures over the life course (Ben-Shlomo and Kuh, 2002, Ben-Shlomo et al., 2014), an obvious alternative being that it is early adolescence, rather than early childhood, when poverty will have most impact on adolescent health. Findings of this nature would suggest that income acts not so much via developmental but through more proximal mechanisms (e.g. by restricting or enabling access to health relevant social and economic resources; Galobardes et al., 2006, Link and Phelan, 1995, Sawyer et al., 2012). These mechanisms might include access to goods and services such as health care, medication, nutritious food, and leisure activities (providing opportunities for exercise or social engagement). Economic stress on parents may also reduce parenting quality (Conger and Elder, 1994, McLeod and Shanahan, 1993). To the extent that these health resources have relatively immediate and short-term impacts on adolescent health and behaviour, we might expect more proximal measures of income, such as in early adolescence, to exhibit the strongest associations. Thus, early adolescence may manifest as either a critical or sensitive period instead of early childhood.

Alternatively, timing may matter less than the duration or sequencing of exposure to poverty (Lee, 2014, Mishra et al., 2009, Wagmiller et al., 2006). Accumulation models posit that the effects of low income in early life would be no more important than the effects of low income at other stages of childhood, and low income at any stage would be associated cumulatively with health outcomes in adolescence. If the health relevant resources associated with income have long-term or lasting impacts on health or behaviours, then we might expect evidence to support an accumulation model as these longer-term impacts accumulate with increased exposure. These long-term impacts could be seen as independent of exposure timing in contrast to those posited for the early childhood critical/sensitive period models, which are tied to developmental processes in early life.

Social mobility models differ by emphasising the sequencing of exposure and the direction of change in SES over time, i.e. especially detrimental effects might be associated with particular patterns of exposure, such as moving into poverty or persistent poverty (Ben-Shlomo et al., 2014, McLeod and Shanahan, 1993, Mishra et al., 2009). The timing of moves in or out of poverty in early vs later childhood could also matter depending on the developmental or proximal mechanisms involved.

Determining which of these varied models best fits the data is important because they have different implications for the timing of intervention (e.g. early childhood vs early adolescence) and the type of mechanisms involved (e.g. developmental processes vs proximal resources), but can be challenging as aspects of the timing, duration and sequencing of exposure may be conflated (Lee, 2014, Mishra et al., 2009). The structured life course approach (Mishra et al., 2009, Smith et al., 2015) in which a closed set of hypotheses is proposed and tests conducted to identify best-fitting hypotheses can be a useful method for differentiating between models of life course exposure. Competing *a priori* hypotheses are encoded in a set of variables, which are then separately added to a regression model predicting an outcome of interest: those variables that give the best improvements in model fit indicate which hypotheses best fit the data. We use this method to compare various models for the effect of poverty in childhood on adolescent health and health behaviours.

Importantly, the impact of poverty timing may vary across country-level policy regimes with differing degrees of decommodification, i.e. the extent to which a socially acceptable standard of living can be maintained, regardless of market performance (or SES; Bambra, 2005, Esping-Andersen, 1987). Even within broadly similar regimes, social policies may be enacted in heterogeneous ways to promote health and aid families, and this heterogeneity could condition the health implications of growing up in poverty. The US and the UK represent a potentially informative contrast. Classifications based on decommodification via cash transfers group them closely together as ‘liberal’ regimes where benefits are modest, strictly controlled by entitlement criteria, and often stigmatised (Bambra, 2005, Esping-Andersen, 1990). On the other hand, a similarly-constructed classification based on decommodification of health care services places the US and UK at opposite ends of the distribution of 18 countries examined, with health care being very dependent on SES in US, where public healthcare coverage has been very limited, often requiring co-payments for use of healthcare services (van Doorslaer and Wagstaff, 1992), and much less so in the UK, with its universal-access national health service (NHS; Bambra, 2005). Thus, although somewhat similar in terms of cash transfers, the US and UK differ markedly in access to health care. Contrasting adolescents' health outcomes (whether physical or mental) with health behaviours such as smoking may therefore be particularly informative. We would expect smaller accumulative or proximal effects of poverty on physical and mental health where health care is less dependent on income (i.e. in the UK compared to the US), but such differences should be less pronounced for a health behaviour such as smoking, which we posit would be relatively independent of health care regime.

This study, therefore, uses a structured life course approach to compare the observed effects of exposure to poverty at different stages of childhood on adolescent smoking and physical and mental health in the US and the UK. The structured life course approach contrasts different hypotheses regarding life course exposures (e.g. accumulation or sensitive periods in early childhood or early adolescence). Another hypothesis is that, for physical and mental health but not smoking, there will be less evidence for accumulation or proximal effects of poverty in the UK compared to the US. This pattern may be indicated by variables representing these hypotheses ranking less well in terms of explaining the data in the UK than in the US, or having smaller effect sizes.

## 2 Methods

### 2.1 Sample

UK data were from the British Household Panel Survey (BHPS) and its successor, Understanding Society (University of Essex, 2010, University of Essex, 2016). The annual BHPS has followed a sample of UK households from 1991 to 2008, with continued follow-up from 2010 to 2013 within Understanding Society. We refer to these two studies collectively as the UK Household Longitudinal Survey (UKHLS). We focus on 16-year-olds surveyed from 2007 onwards (n = 1204; born 1991–1997), as the survey potentially had annual records (relating to their mothers) back to birth for these children.

US respondents were from the National Longitudinal Survey of Youth 1979 Child/Young Adult sample (NLSY79-YA; Bureau of Labor Statistics, 2017). These respondents are the biological children of the mothers in the National Longitudinal Survey of Youth 1979 (NLSY79; Bureau of Labor Statistics & US Department of Labor, 2014). Information on the biological mothers and the respondents' income history comes from the NLSY79. Surveys were annual up to 1994 and biennial thereafter. We focus on respondents who were born between 1979 and 1996 (so that mothers were eligible to be surveyed throughout the respondents’ childhoods; n = 9408).

### 2.2 Measures

#### 2.2.1 Outcomes

Outcomes were measured at age 16 in UKHLS and at age 15 or 16 for NLSY79-YA (depending on when they were surveyed). Smoking was self-reported as any current smoking in both surveys. In 2007/2008 BHPS respondents were asked, ‘Does your health in any way limit your activities compared to most people of your age?’ (yes/no) and ‘Does your health limit the type of work or the amount of work you can do? (yes/no). Understanding Society respondents were asked each year if their physical health limited the kind of work or other daily activities they did in the past 4 weeks (5 categories; all of the time-none of the time). In the NLSY79-YA children were asked if their health prevented them from working or going to school, excluding temporary limitations due to pregnancy. Any limitation was coded as health limitation. For mental health, the UKHLS utilised the 12-item General Health Questionnaire (GHQ; Goldberg and Williams, 1988) and scores of three or more were coded as indicating possible psychiatric distress (Banks, 1983). Respondents in the NLSY79-YA responded to the short-form Center for Epidemiological Studies Depression scale (CES-D) and psychiatric distress was indicated by scores of 8 or more (Levine, 2013, Radloff, 1977).

### 2.3 Income and exposure variables

Each survey included measures of net annual household income. Annual income measures were equivalised for household size and poverty was coded as less than 60% of the national median equivalised income for that year using publicly available historical data (Department for Work and Pensions, 2015, United States Census Bureau, 2016). Inflation was adjusted for by defining poverty in comparison to the national median within the year of observation. Table 1 shows 10 (mostly binary) variables encoding different hypotheses regarding exposure to poverty across three periods of childhood referred to respectively as early life (ages 0–5), mid-childhood (ages 6–10), and early adolescence (ages 11–15). Critical period hypotheses are represented respectively by binary indicators of poverty in early life, mid-childhood and early adolescence, whereas the accumulation hypothesis is represented by a cumulative sum of the periods in which exposure to poverty has occurred. The remaining variables represent other possible alternative hypotheses that emphasise social mobility, persistent poverty, or a threshold-style effect, whereby any exposure to poverty at all is associated with a uniform difference in the outcome. Sensitive periods can be represented by combining variables, e.g. by combining one of the critical period variables with the cumulative exposure variable, or simply by combining two or more of the critical period variables.

**Table 1:** Poverty exposure variable definitions.

Variable	Definition
Early Life Poverty	1 = in poverty at any year ages 0-5
	0 = no poverty ages 0-5
Mid-Childhood Poverty	1 = in poverty at any year ages 6-10
	0 = no poverty ages 6-10
Early Adolescent Poverty	1 = in poverty at any year ages 11-15
	0 = no poverty ages 11-15
Cumulative Poverty	Sum of early life, mid-childhood and early adolescent variables (range: 0–3)
Early Upward Mobility	1 = poverty in early life but no poverty in mid-childhood
	0 = all else
Early Downward Mobility	1 = no poverty in early life but poverty in mid-childhood
	0 = all else
Later Upward Mobility	1 = poverty in mid-childhood but no poverty in early adolescence
	0 = all else
Later Downward Mobility	1 = no poverty in mid-childhood but poverty in early adolescence
	0 = all else
Persistent Poverty	1 = poverty in early life, mid-childhood and early adolescence
	0 = all else
Any Poverty	1 = poverty in early life, mid-childhood or early adolescence
	0 = all else

### 2.4 Covariates

The following covariates were included for both countries: gender, cohort (i.e. year of birth), and mother's years of education and mental health (UK: GHQ; US: CES-D short-form) at birth. UK analyses were additionally adjusted for mother's ethnicity (0 = white, 1 = non-white), smoking status (any current smoking vs none), mother's age, and family structure (cohabiting couples vs single parents) at age 0, while US analyses were adjusted for mother's nativity status (US vs foreign-born), child's race (black, Hispanic or other), whether the mother smoked prior to pregnancy, and family structure at birth (married co-resident couples vs all others), and were weighted for mother's age (see below).

### 2.5 Analysis

For each outcome variable, modelling proceeded as follows: First, we estimated a logistic regression model predicting the outcome with all covariates and no poverty variables. Second, each poverty variable was separately added to the model, and improvement in fit was assessed with a Wald test. All variables that produced a significant improvement as assessed by the Wald test (p < 0.05) were then compared using the Bayesian Information Criterion (BIC). The variable showing the greatest improvement in fit as measured by the BIC was retained and the process was repeated with all the remaining poverty variables (to see if a combination of poverty variables gave better fit) until no further variables produced significant improvements to the model. Standard errors were adjusted for the fact that respondents could be clustered within families. As a final step, we used z-tests (Clogg et al., 1995) to compare the magnitude of poverty coefficients from models with the best-fitting variables across the two countries.

Multiple imputation (25 datasets) and weighting adjusted for missing data (Seaman et al., 2012). Both surveys used cross-sectional sampling weights for the year in which a respondent turned 16 to account for sample attrition and over-sampling. For NLSY79-YA cross-sectional sampling weights were additionally multiplied with weights accounting for the fact that younger cohorts were disproportionately born to younger mothers. These weights were created by predicting the probability that a mother would be a teenager at their child's birth using the following variables: own mother's age at birth, race, parent's education, family structure, region of residence, immigrant generational status, and number of siblings. Imputation models were unconstrained two-level models with years nested within persons (Asparouhov and Muthén, 2010) and included all the covariates, the analysis weights, and poverty status at each year. Poverty status and annually measured covariates (family structure, maternal smoking and maternal mental health) were included at the within-person level, whilst all other variables were included at the between-person level. Descriptive statistics and model results were weighted and averaged across the 25 datasets.

## 3 Results

Table 2 shows descriptive statistics for both samples. Both samples had similar rates of poverty in early life, but the UK had higher rates of upward mobility and lower rates of poverty in mid-childhood and early adolescence. Thus, although similar proportions had any experience of poverty, persistent poverty was considerably less common in the UK than in the US. Rates of smoking were similar across countries, but rates of health limitations and poor mental health were higher in the UK than in the US, although this difference does not necessarily indicate worse health in the UK given that measurement differed.

**Table 2:** Descriptive statistics for analysis samples.

Variable	NLSY79-YA (n = 9408)		UKHLS (n = 1204)		P-Value^a^
	Categories	%	Categories (where different)	%	
**Poverty Exposure**					
Early Life Poverty		65.2		63.4	0.211
Mid-Childhood Poverty		58.6		49.2	<0.001
Early Adolescent Poverty		55.1		45.2	<0.001
Early upward mobility		15.9		22.5	<0.001
Early downward mobility		9.2		8.3	0.338
Later upward mobility		13.5		18.5	<0.001
Later downward mobility		10.1		14.6	<0.001
Cumulative Poverty (# of periods of poverty)	0	21.1		21.8	<0.001
	1	19.9		24.8	
	2	18.4		27.2	
	3	40.7		26.2	
Persistent poverty		40.7		26.2	<0.001
Any poverty		79.2		78.2	0.452
**Adolescent Outcomes**					
Current smoker		12.1		12.5	0.676
Health limits activities		3.6		10.6	<0.001
Poor mental health^a^		16.2		22.4	<0.001
**Covariates**					
Gender	Male	51.4		51.9	0.759
	Female	48.6		48.1	
Race/Ethnicity	Hispanic	7.7	White	95.9	n/a
	Black	16.2	Other	4.1	
	Other	76.1			
Family structure at birth	Married Couple	74.8	Couple	87.8	<0.001
	Other	25.2	Single	12.2	
Year of birth	1979–1984	33.4	1991	23.9	n/a
	1985–1990	39.8	1992	19.7	
	1991–1996	26.8	1994	17.9	
			1995	13.8	
			1996	11.5	
			1997	13.1	
Mother's age at birth	15–19	6.9		6.1	<0.001
	20–24	31.9		18.3	
	25–29	35.2		29.7	
	30–34	22.1		28.3	
	35+	3.9		17.6	
Mother's education (years)	11 or less	5.8		45.4	<0.001
	12–14	70.4		31.5	
	15 or more	23.9		23.1	
Maternal Smoking^b^		32.5		25.3	<0.001
Maternal mental health difficulties^c^		22.3		32.9	<0.001
Mother foreign born		4.1		n/a	n/a

a Chi-Square test for difference.

b In NLSY79-YA mothers self-reported whether they had smoked prior to pregnancy. In UKHLS mothers self-reported their smoking status when the child was aged 0.

c Poor mental health was indicated by CES-D short-form scores of 8 + in NLSY79-YA and by GHQ scores of 3 + in UKHLS.

Table 3, Table 4 compare BIC statistics and *p*-values for the poverty variables from various models for the US and UK data respectively, as well as odds ratios (ORs) and 95% confidence intervals (95% CI) for the best fitting poverty variables. The first row of each table shows the basic models with all covariates, and the subsequent rows show model fit after adding each poverty variable separately. Cumulative poverty offered the best fit for smoking in the US and for health limitations in the UK, but persistent poverty gave the best fit for smoking in the UK and for health limitations in the US. For smoking and health limitations, model fit was similar for cumulative and persistent poverty; they were in all cases the two best fitting variables. No poverty variable significantly predicted poor mental health in either sample. After retaining cumulative poverty in the model, early life poverty further improved the model for smoking in the US sample (see Table 3). No other poverty variables made any improvements to fit after retaining the first best-fitting variable.

**Table 3:** Model fit statistics from US data.

Model	Current Smoking		Health Limitations		Poor Mental Health	
	BIC	P-Value	BIC	P-Value	BIC	P-Value
Basic^a^	6085.1	–	2945.5	–	8262.0^b^	–
Early life poverty	6079.6	0.039	2945.8	0.105	8267.0	0.251
Mid-childhood poverty	6084.3	0.082	2945.0	0.056	8270.9	0.972
Early adolescent poverty	6082.8	0.067	2942.2	0.034	8269.9	0.658
Early upward mobility	6090.1	0.308	2950.1	0.222	8269.9	0.637
Early downward mobility	6092.8	0.124	2949.6	0.257	8267.2	0.354
Late upward mobility	6092.8	0.688	2950.5	0.315	8269.5	0.620
Late downward mobility	6093.6	0.801	2952.4	0.435	8270.2	0.986
Cumulative poverty	6073.2	0.010	2936.6	0.014	8269.5	0.447
Persistent poverty	6074.6	0.010	2928.5^b^	0.002	8266.9	0.201
Any poverty	6089.7	0.356	2952.2	0.445	8270.5	0.782

Cumulative + Early life	6072.4^b^		n/a			
Cumulative poverty		0.009				
Early Life poverty		0.009				

**Best Fitting Variables**						
	**OR**	**95% CI**	**OR**	**95% CI**	**OR**	**95% CI**
Early life poverty	1.14	1.03–1.25	–	–	–	–
Cumulative poverty^c^	1.14	1.03–1.25	–	–	–	–
Persistent poverty	–	–	1.98	1.30–3.03	–	–

a Adjusted for gender, race, family structure at birth, year of birth, mother's education, maternal smoking prior to pregnancy, maternal mental health at birth, and whether mother was US or foreign born.

b Best fitting model for this outcome.

c OR indicates increase in risk associated with each period in which poverty was experienced.

**Table 4:** Model fit statistics from UK data.

Model	Current Smoking		Health Limitations		Poor Mental Health	
	BIC	P-Value	BIC	P-Value	BIC	P-Value
Basic^a^	963.2	–	862.0	–	1288.5^b^	–
Early life poverty	964.9	0.097	859.9	0.039	1294.7	0.815
Mid-childhood poverty	958.5	0.014	862.1	0.037	1294.4	0.469
Early adolescent poverty	962.8	0.046	859.5	0.037	1295.3	0.861
Early upward mobility	965.5	0.159	868.6	0.849	1292.6	0.720
Early downward mobility	969.3	0.561	868.4	0.899	1292.6	0.217
Late upward mobility	969.6	0.845	868.4	0.809	1295.0	0.347
Late downward mobility	967.9	0.410	868.4	0.874	1292.9	0.970
Cumulative poverty	954.8	0.004	853.0^b^	0.003	1294.9	0.290
Persistent poverty	953.2^b^	0.002	859.2	0.020	1292.8	0.231
Any poverty	966.1	0.168	859.4	0.027	1294.4	0.672

**Best Fitting Variables**						
	**OR**	**95% CI**	**OR**	**95% CI**	**OR**	**95% CI**
Cumulative poverty^c^	–	–	1.46	1.14–1.87	–	–
Persistent poverty	2.20	1.32–3.65	–	–	–	–

a Adjusted for gender, mother's ethnicity, family structure at birth, year of birth, mother's age at birth, mother's education, maternal smoking and maternal mental health at age 0.

b Best fitting model for this outcome.

c OR indicates increase in risk associated with each period in which poverty was experienced.

Table 5 contains cross-country comparisons of coefficients from models with cumulative and persistent poverty variables for both current smoking and health limitations and one combining cumulative and early life poverty for current smoking (mental health was not assessed at this stage because there were no significant associations between mental health and poverty). Although poverty coefficients were mostly larger in magnitude in the UK than in the US, none differed significantly between the two countries. This pattern even extended to early life poverty and smoking, where the US coefficient differed significantly from zero while that for the UK was in the opposite direction and was statistically insignificant.

**Table 5:** Comparison of model coefficients between US and UK data.

Model		US^a^			UK^b^			Comparison	
		Beta	SE	P-Value	Beta	SE	P-Value	Z	P-Value
*Current Smoking*									
Cumulative exposure only		0.164	0.064	0.010	0.346	0.119	0.004	−1.347	0.178
Persistent poverty only		0.336	0.131	0.010	0.787	0.259	0.002	−1.554	0.120
Early Life Sensitive Period	Cumulative exposure	0.127	0.049	0.009	0.391	0.158	0.013	−1.596	0.110
	Early life poverty	0.127	0.049	0.009	−0.152	0.367	0.679	0.754	0.451

*Health Limitations*									
Cumulative exposure only		0.258	0.104	0.014	0.378	0.126	0.003	−0.734	0.462
Persistent poverty only		0.684	0.217	0.002	0.657	0.281	0.019	0.076	0.939

a Adjusted for gender, race, family structure at birth, year of birth, mother's education, maternal smoking prior to pregnancy, maternal mental health at birth, and whether mother was US or foreign born.

b Adjusted for gender, mother's ethnicity, family structure at birth, year of birth, mother's age at birth, mother's education, maternal smoking and maternal mental health at age 0.

## 4 Discussion

### 4.1 Principal findings

This study is the first to use a structured life course approach to examine associations between different patterns of childhood exposure to poverty and adolescent smoking and physical and mental health in the US and the UK. Poverty was more persistent in the US than in the UK, and, in both countries, associated with higher rates of adolescent smoking and health limitations, but not associated with adolescent mental health. For smoking and health limitations, variables representing cumulative and persistent poverty provided better fit to the data than variables representing alternative patterns of poverty exposure. Cumulative poverty offered the best fit for health limitations in the UK and for smoking in the US, whilst persistent poverty gave the best fit for smoking in the UK and for health limitations in the US. After accounting for cumulative poverty exposure, early life poverty was additionally associated with higher rates of smoking in the US, suggesting a sensitive period in early life. There was little evidence to support the hypothesis that cumulative or proximal exposures had weaker effects in the UK than in the US; associations between outcomes and poverty within the two studies were remarkably similar (and were, if anything, larger in magnitude in the UK than the US).

### 4.2 Strengths and weaknesses

In contrast to methods that aim to classify observed exposure trajectories (e.g. see Lee, 2014, Wagmiller et al., 2006), the structured life course approach utilised here has enabled comparison of *a priori* hypotheses regarding life course exposure to poverty and adolescent health and behaviour, and identified that accumulation hypotheses generally fit the data better than hypotheses regarding social mobility and sensitive or critical periods in early life or adolescence (only for smoking in the US did an early life sensitive period optimise model fit). A limitation of this approach is that when competing variables offer close or comparable levels of model fit, there is not strong evidence for one hypothesis over the other. We experienced this issue here with cumulative and persistent poverty variables giving comparable improvements in fit, particularly for smoking (in both countries).

These analyses adjusted for a range of potential confounders (such as parental smoking or mental health), but they were generally only included at baseline, so models were not adjusted for later changes in these factors, which may or may not have been caused by earlier poverty. Marginal structural models represent a promising method of adjusting for such time-varying confounding in future research (Lee, 2014). Further, since health was not measured through childhood, it is unclear whether the adolescent health outcomes represent health problems emerging in adolescence as a result of predictors or long-standing problems that developed earlier in childhood, and potentially contributed to family income poverty (though for smoking at least it is unlikely that the habit developed in early or mid-childhood). There were some minor differences in the confounding variables employed for the US and UK analyses, but the results are nevertheless reasonably consistent despite differences in measurement and confounding structures, which strengthens the case for the associations with poverty being causal. Indeed, it has been suggested that socioeconomic position may be a ‘fundamental cause’ of health, with persistent and enduring effects related to the stratification of access to resources, despite variations across time and place in the specific mechanisms or resources that link disadvantage to poor health (Link and Phelan, 1995).

In comparing the US and the UK, we have drawn on classifications of welfare regimes that emphasise decommodification in terms of cash transfers (Esping-Andersen, 1990) and health care services (Bambra, 2005), suggesting that the two countries have similar approaches in terms of cash transfers, but very different approaches to health care services. This is probably an over-simplification. These classifications are based on relatively old data (i.e. from 1980 whilst our data spans 1979–2012 for the US and 1991–2013 for the UK), whereas country-level approaches to decommodification may change over time as different policies are introduced or retired (Bambra, 2007). Additionally, these classifications may mask heterogeneity in policies, even where countries were classified as similar (Bambra, 2005, Bambra, 2007). The predicted differences did not emerge, as associations between poverty and health outcomes were generally similar in the US and UK. The persistence of poverty, however, did appear to be greater within the US. This difference may reflect variations in welfare policies between the two countries over the time periods studied, which are not represented in the classification used. Further, we observed an early life sensitive period for smoking in the US, but not in the UK. This difference may have been due to differences in sample size, with the US study having more power to detect such effects (the UK estimate for this coefficient was in the opposite direction, but a z-test comparison still did not indicate a significant difference).

### 4.3 Comparisons with other literature

We are not aware of other studies examining the timing of poverty in childhood in relation to adolescent health outcomes, but others have examined socioeconomic inequalities in adolescent health. Findings for physical health are inconsistent: some show inequalities (Chen et al., 2006, Emerson et al., 2005), while others do not (Siahpush and Singh, 2000, Sweeting et al., 2016, West et al., 1990). If cumulative or persistent poverty is important, inconsistencies in prior research may be due to insufficient attention to the life course pattern of exposure. Socioeconomic disadvantages in risk for smoking, as observed here, are a consistent finding (Hanson and Chen, 2007), while the lack of association between poverty and mental health is consistent with some (Miech and Shanahan, 2000, Siahpush and Singh, 2000, Sweeting et al., 2016, West et al., 1990) but not all (Joinson et al., 2016) previous research. An earlier study using the children of the NLSY79 did show associations between persistent poverty and mental health measured in mid-childhood (McLeod and Shanahan, 1993).

Duncan et al. (1998) examined poverty exposure in early life, mid-childhood and early adolescence in relation to educational outcomes in the US. Their findings indicated that poverty in early life had a particularly strong effect on educational outcomes (i.e. a sensitive period), contrasting with the emphasis our findings place on cumulative or persistent poverty. The educational system is so highly cumulative that starting points are strong predictors of later progress, which may be why early poverty matters more for education than health. More recent studies of poverty exposure trajectories, have emphasised persistent poverty as a strong determinant of educational achievement (Lee, 2014, Wagmiller et al., 2006), in line with our findings for health outcomes.

### 4.4 Meaning and implications

Good fit for cumulative poverty indicates that health risks rise incrementally with longer exposure, whereas good fit for persistent poverty indicates increased risk only for those who experienced poverty within every stage of childhood. Unfortunately, this provides different conclusions as to the most effective timing of intervention. Persistent poverty suggests intervention in early adolescence as it is only at this stage that persistence becomes apparent: some of those in poverty at earlier ages may move out of poverty without intervention. On the other hand, cumulative poverty (or duration of exposure) suggests intervention in early life, as any causal link between poverty in early life and poverty later in childhood will mean an early life intervention has the additional benefit of reducing risk for prolonged exposure (Green and Popham, 2017). Given that persistent and cumulative poverty variables were close in terms of model fit for health limitations and smoking, there is not strong evidence for one over the other for these outcomes. However, it may on balance be best to intervene to raise income in early life. Compared to other stages of childhood, intervention in early life: 1) may have the strongest influence on adolescent health and behaviour if the true causal effect of poverty is cumulative; 2) corresponds with the timing of intervention indicated by some earlier research (e.g. on educational outcomes; Duncan et al., 1998); 3) is further supported by our finding of an early life sensitive period for smoking in the US; and 4) will still be beneficial (if not maximally efficient) if the true causal effect lies with persistent poverty.

With regards to mechanisms, findings did not indicate that developmental processes in early life are especially important, but there was evidence in the US of an early life sensitivity to poverty, suggesting an effect on development (e.g. social, emotional, physical or cognitive) producing an enduring predisposition to smoking not generated by poverty at other stages of life. Cumulative risk models were better supported by the data for physical health and smoking and suggest mechanisms of influence involving long-term, lasting impacts of health relevant resources or stressors associated with income that accumulate over time. For example, the mechanisms linking poverty and physical health may have more to do with long-term exposure to damp and over-crowded housing, than with proximal access to health care and medicines when a health problem occurs. For smoking, the under-lying mechanisms are likely to be related to psychosocial resources as cigarettes cost money so psychosocial mechanisms are needed to explain increased risk among those with fewer material resources (Laaksonen et al., 2005). Thus, the association with cumulative poverty may be more about the cultural availability of smoking as an acceptable behaviour, than about the proximal, physical availability of cigarettes or acute parental monitoring (Michie and Abraham, 2004).

Nevertheless if such cumulative mechanisms increase risk for poor physical health and smoking behaviour, it is curious that they do not also appear to increase risk for poor mental health. One explanation for the lack of association between poverty and mental health is that young people from more affluent backgrounds experience elevated distress levels due to educational pressures during schooling (West and Sweeting, 2003). This explanation would be consistent with research showing that inequalities, with higher distress levels in socioeconomic disadvantage, emerge as young people move into adulthood (and educational pressures presumably recede; Green and Benzeval, 2011, Miech and Shanahan, 2000, Sweeting et al., 2016), and with previous research in this US sample showing associations between persistent poverty and poor mental health in mid-childhood (i.e. before academic anxieties become prominent; McLeod and Shanahan, 1993).

### 4.5 Future research

Considering that the findings of this study emphasise the cumulative effects of poverty, a key question for future research may be whether early life poverty is causally linked to poverty in later childhood, i.e. if you intervene to raise income in early life, will this prevent low income at later stages of childhood? If not, there may be no particular advantage of intervening in early rather than later childhood (Green and Popham, 2017). Also, worthy of further exploration are ways in which the macro social context influences life course patterns of childhood poverty. One of the main differences we observed between the US and the UK was not in the effects of, but in the persistence of poverty. If we could better understand the mechanisms, and ideally social policies, that account for this difference, we could be better prepared to alleviate poverty and its associated health burdens. Additionally, why the early life sensitive period observed in the US was not observed in our UK sample is unclear. This difference may have just been an issue of statistical power, with the UK sample being smaller, but further attempts at replication with larger samples from the UK and other developed countries could confirm whether this is a consistent and generalizable finding.

## 5 Conclusions

Drawing on past longitudinal studies linking poverty to key life course outcomes and comparing two-samples from different countries, this study provides the most rigorous empirical evidence to-date on the timing of exposure to poverty in childhood and adolescent health and behaviour. It compared competing hypotheses of early life or early adolescent critical/sensitive periods, social mobility models, and accumulative risk, finding stronger evidence overall for an accumulation model than for other models in relation to smoking and health limitations on daily activities. Adolescent mental health did not seem associated with poverty exposure. On balance, early life interventions to raise family income or buffer against the deleterious effects of low income may have the best chance of alleviating the adolescent health burdens associated with poverty.

## Conflict of interest

None.
